# Formation and Resuscitation of Viable but Nonculturable *Salmonella typhi*

**DOI:** 10.1155/2013/907170

**Published:** 2012-12-26

**Authors:** Bin Zeng, Guozhong Zhao, Xiaohong Cao, Zhen Yang, Chunling Wang, Lihua Hou

**Affiliations:** ^1^School of Life Sciences, Jiangxi Science and Technology Normal University, Nanchang 330013, China; ^2^Key Laboratory of Food Nutrition and Safety, Ministry of Education, Tianjin University of Science and Technology, Tianjin 300457, China

## Abstract

*Salmonella typhi* is a pathogen that causes the human disease of typhoid fever. The aim of this study was to investigate the viable but nonculturable (VBNC) state of *S. typhi*. Some samples were stimulated at 4°C or −20°C, while others were induced by different concentrations of CuSO_4_. Total cell counts remained constant throughout several days by acridine orange direct counting; however, plate counts declined to undetectable levels within 48 hours by plate counting at −20°C. The direct viable counts remained fairly constant at this level by direct viable counting. Carbon and nitrogen materials slowly decreased which indicated that a large population of cells existed in the VBNC state and entered the VBNC state in response to exposure to 0.01 or 0.015 mmol/L CuSO_4_ for more than 14 or 12 days, respectively. Adding 3% Tween 20 or 1% catalase enabled cells to become culturable again, with resuscitation times of 48 h and 24 h, respectively. The atomic force microscope results showed that cells gradually changed in shape from short rods to coccoids, and decreased in size when they entered the VBNC state. Further animal experiments suggested that resuscitated cells might regain pathogenicity.

## 1. Introduction

*Salmonella enterica* serovar Typhi, a type of Gram-negative bacterium, is the causative pathogen of typhoid fever, a human disease [1, 2]. Salmonellosis is a significant cause of diarrheal illness in humans, causing ~1.4 million illnesses and an average of 600 deaths annually in the United States [3]. Previous investigations have determined that human infections principally result from the ingestion of meat, eggs, corn products, fruit, vegetables and drinking water [4–7]. *Salmonella* is routinely detected by the use of enrichment and selective media, but large differences have been consistently reported between results from cultivation and total direct microscopic counts. However, little is known about the reasons for these differences, and the causes of disease occurrence remain elusive.

It has been reported that some strains of bacteria especially the *Salmonella* [8–10] enter the viable but nonculturable state (VBNC) when they encounter environmental stresses, such as low temperatures, oligotrophic conditions, infiltration press, and biocides including heavy metals and ultraviolet radiation [11, 12]. VBNC bacteria are organisms that fail to grow and develop colonies on media, but their metabolic activity capabilities indicate that they are still alive [13]. Bacteria in the VBNC state show changes in cell physiology and morphology [14]. Commercially bottled drinking water is at risk of harboring multiple VBNC bacterial strains, as it may contain copper-based biocides [15] and be stored at low temperatures without nutrients for bacterial growth.

Some experiments have demonstrated that VBNC-state bacteria still show metabolic activities [16, 17], but cannot be cultured under standard procedures. Glucose content [18] and (NH_4_)_2_SO_4_ concentration [19] were used to measure the metabolic activity of bacteria. The VBNC-state Salmonella [8] or several other VBNC-state bacteria [20, 21] have be resuscitated with a simple temperature increase and adding catalase [22] or Tween 20 [23]. It is reported that supplemented catalase acted by preventing the accumulation of hydrogen peroxide in VBNC cells and Tween 20 made up for a deficiency of carbon [22, 24]. Pathogens in the VBNC state may retain their pathogenicity, and the resuscitation of these pathogenic cells has recently become an important field of investigation as a result of the public health implications for both humans and animals.

VBNC-state *S. typhi* can exist in meat, eggs, corn products, fruit, vegetables, and drinking water, and are therefore an obvious source of potential illness in humans. To our knowledge, little is known about the existence of VBNC *S. typhi* in food and drinking water, and whether they can be resuscitated in the human. In the present study, we sought to investigate whether *S. typhi* could enter the VBNC state under starvation conditions at low temperatures or after copper ion stimulation, and what conditions could resuscitate VBNC forms. This study enhances our ability to detect pathogenic bacteria in our drinking water and is a highly important avenue of preventative public health research. In addition, we demonstrate a novel method for the culture collection of these bacteria.

## 2. Materials and Methods

### 2.1. Bacterial Strain and Growth Conditions

*S. typhi* was obtained from the Bacterial Culture and Collection Center of Tianjin University of Science and Technology, China, for use in the microcosm experiments. The strain was stocked in nutrient broth with 10% (v/v) glycerol at −80°C.

### 2.2. Preparation of the Microcosm

The microcosm was prepared by filtering bottled drinking water through a 0.22-*μ*m Millipore filter, after which it was sterilized by autoclaving (pH, 7.9 ± 0.1). It has been reported that bacteria can easily enter the VBNC state during the logarithmic phase [25]. Bacterial cell cultures in the logarithmic phase were therefore harvested by centrifugation at 5,000 rpm for 10 min at 4°C and washed twice with autoclaved saline water (1 M NaCl solution, 0.9% bw) which may prevent eventual cell damage and cell death. Then, the washed cells were filtered through a 0.22-*μ*m Millipore membrane filter and resuspended in the sterilized water at a final density of 10^7^ CFU/mL [15]. The inoculated microcosm was maintained at 4°C, and the culturable cell populations were counted with a gradient dilution technique and determined on nutrient agar plate cultures at 37°C. The experiments were conducted in triplicate.

### 2.3. Nonculturability and Viability Assays

The microcosm water system at a final concentration of approximately 10^7^ CFU/mL was used to starve *S. typhi* cells. Samples were then left in the dark for 100 d at 4°C or −20°C, and some ones were treated with different concentrations of CuSO_4_ (0.005, 0.01, 0.015, and 0.025 mmol/L) at room temperature. To determine the number of culturable cells, samples were plated on nutrient agar plates at 37°C for 12 h. All these studies were performed in triplicate.

The number of total cells in the microcosm samples was determined by the acridine orange direct count (AODC) method [26]. Samples were serially diluted and fixed with formalin (2% v/v), stained with acridine orange (0.01% w/v) for 2 min and then examined under a Nikon 90i fluorescence microscope (Nikon, Japan) in a dark room. The excitation light filter used was 480 nm, and the fluorescence filter was 510 nm.

Kogure first used the direct viable count (DVC) method in 1979 [27]. Yeast extract (0.025% w/v) and nalidixic acid (0.002% w/v) were added and then incubated at 37°C for 6 h before acridine orange staining. Cells that were elongated to at least twice the length of the AODC cells were marked as viable. More than three fields of view were counted in this study.

### 2.4. Metabolic Ability of VBNC Cells

The metabolic activity of VBNC cells could not be measured simply. This experiment was performed at room temperature about 48 h, and the basic nutrition, glucose content and (NH_4_)_2_SO_4_ concentration as the only carbon and nitrogen sources were added into the sterilized and filtered bottled drinking water. This medium (Glucose 0.6%; (NH_4_)_2_SO_4_ 0.2%; MgSO_4_·7H_2_O 0.02%; K_2_HPO_4_ 0.4%; KH_2_PO_4_ 0.6%) was used to measure the metabolic ability of normal and VBNC cells. Normal cells and the VBNC cells (<0.1 CFU/mL cells determined by plate counting) were treated at −20°C or induced by CuSO_4_ of 0.025 mmol/L. The glucose content was determined by the consumption of Fehling's solution [28, 29]. The (NH_4_)_2_SO_4_ was measured by ion chromatography, where the flow speed and pressure were 0.81 mL/min and 1,546 psi [30].

### 2.5. Resuscitation of the VBNC Cells

Nutrient broth was filtered through a 0.22 *μ*m Millipore filter and sterilized by autoclaving. 20 *μ*L of the VBNC cells (<0.1 CFU/mL) in this study were inoculated in the 10 mL of nutrient broth. 10 mL of the nutrient broth with 20 *μ*L of sterilized water (the control) and the nutrient broth sample of VBNC cells were resuscitated by these methods: temperature increase [31], or the addition of nutrition like catalase (3500 units/mg, 1% w/v) [31] and Tween 20 (0.5%, 1%, 3%, and 5% v/v) [23].

Resuscitation by a temperature upshift was performed as follows: 10 mL of the sample was removed from the microcosm and initially incubated at 10°C, and then the culture temperature was increased by 5°C every 60 min. The culturability of these 10 mL samples was determined by plating the cells on the nutrient agar plates. 2 mL of the sample was cultured on one plate.

To resuscitate VBNC cells by the addition of nutrition, catalase or Tween 20 was added to the samples. The samples were then incubated at 37°C and culturability was determined by plating. All experiments described here were performed in triplicate.

### 2.6. Atomic Force Microscope (AFM)

The normal cells and VBNC cells were harvested by centrifugation at 10,000 rpm for 10 min. Then, 1 mL sterile PBS was added and shaken three times. The sample was dripped onto a mica tablet and dried at room temperature. Finally, the mica tablets were examined with an AFM.

### 2.7. Virulence Assay

Healthy Kunming mouse [32] which is one of the most important strains of mice widely used in medical (3 months old, 18–22 g) was used to investigate the retention of virulence in both VBNC cells and resuscitated cells. Thirty mice were randomly divided into three groups, each consisting of 10 animals. One group was the control and was administered 0.1 mL of the autoclaved saline water, while the other two groups were administered 0.1 mL of the microcosm of either the VBNCs or the resuscitated cells (10^6^ CFU/mL) by lung lavage every day. *S. typhi* is the pathogen which is responsible for the diarrheal disease [33]. It is always associated with contaminated food or water [6, 34]. The cause of the diarrheal illness in mice was confirmed by re-isolating bacteria from the ascitic fluid after sacrifice.

## 3. Results

### 3.1. VBNC-State *S. typhi*

**Figure 1:**
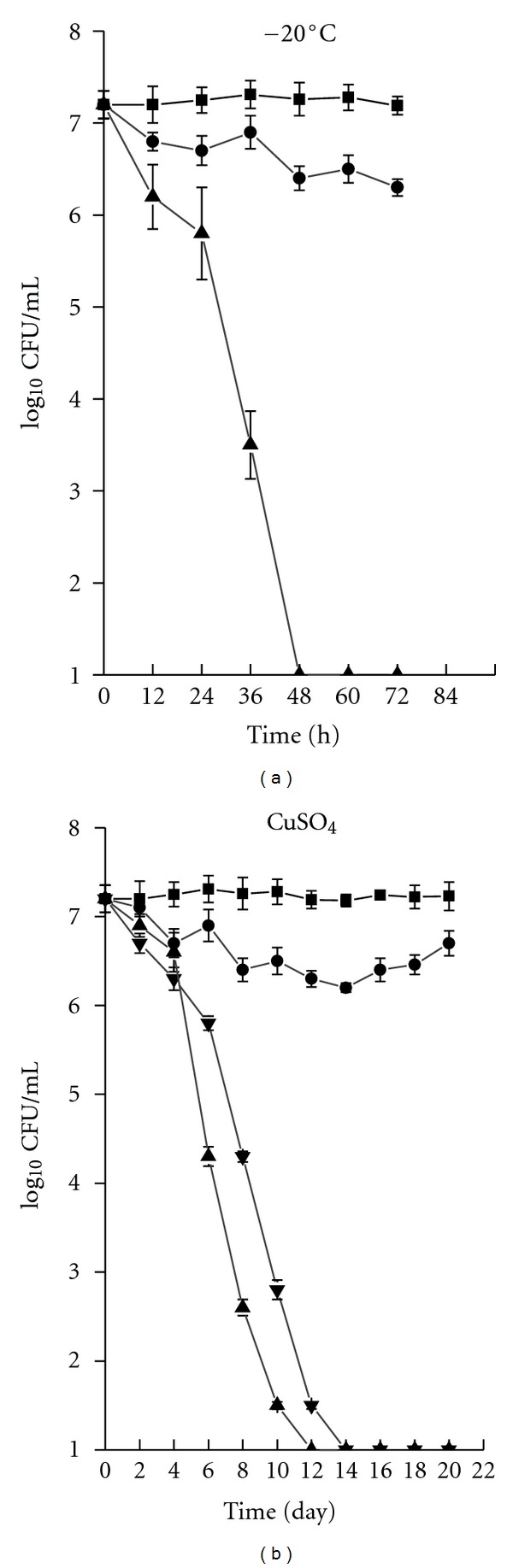
(a) Entry of *S. typhi* into the VBNC state in a microcosm at −20°C, as determined by AODC (■), DVC (●) and plate counting (▲) methods. (b) Entry of *S. typhi* into the VBNC state in a microcosm stimulated by CuSO_4_ at concentrations of 0.01 mmol/L (*▼*) and 0.015 mmol/L (▲) by plate counting, AODC (■) and DVC (●).

VBNC cells could not form after incubation at 4°C for 120 days. The number of bacterial colonies decreased from 2.3 × 10^7^ CFU/mL to 10^6^ CFU/mL over 120 days, and they could still be cultured (data not shown). A much longer time maybe needed to show that cells could enter the VBNC state 4°C.* S. typhi* entered the VBNC state within 48 h of incubation in the bottled drinking water microcosm at −20°C (Figure 1(a)).

Different concentrations of CuSO_4_ were added to the microcosm at room temperature to obtain the VBNC cells. The results were evaluated by plate counting. We found that the bacterial colonies on the agar plate disappeared after 14 and 12 days when the concentrations of CuSO_4_ were 0.01 and 0.015 mmol/L (Figure 1(b)), respectively, but not for the 0.005 and 0.025 mmol/L CuSO_4_. During this period, all total direct counts were not obviously changed. Furthermore, the concentration of active cells as demonstrated by DVC staining decreased slightly to 10^6^ CFU/mL. These results indicated that many cells entered the VBNC state under these conditions.

### 3.2. Morphological Changes of VBNC Cells

**Figure 2:**
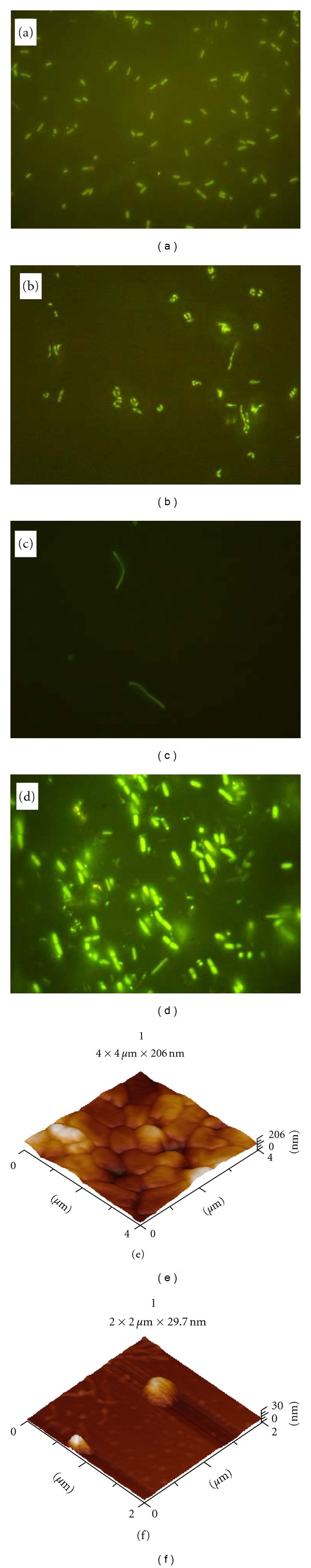
Morphological characteristics of S. typhi under a Nikon 90i fluorescence microscope (a–d) (magnification, 1,000×) or an atomic force microscope (e, f). (a) Normal cells, (b) VBNC cells, (c) VBNC cells after absorbing added nutrition, (d) resuscitated cells, (e) normal cells by AFM, and (f) VBNC cells by AFM.

Significant variations were observed between the VBNC cells and normal cells under fluorescence microscopy by the AODC method (Figures 2(a) and 2(b)). Normal cells were short rods that were dispersed randomly. However, the VBNC cells were smaller than the normal ones and some had assembled together. After nutrition was added, such as yeast extract, the VBNC cells (Figure 2(c)) were shown to become elongated and were defined as viable according to the DVC method. We also observed cells under an AFM and found that the shape of VBNC cells had changed from short rods to coccoids; simultaneously, the cell size also decreased (Figures 2(e) and 2(f)). The average size of the normal cells was 2 × 1 *μ*m (length × width) and the volume is about 1.57 *μ*m^3^, while the radius of the VBNC cells was approximately 0.5 *μ*m and the volume is about 0.785 *μ*m^3^ which is the half of normal cell volume (Figures 2(e) and 2(f)). The resuscitated cells (Figure 2(d)) showed no morphological differences compared with normal cells (Figure 2(d) was digitally magnified about 2 times).

### 3.3. Metabolic Ability of VBNC Cells

**Figure 3:**
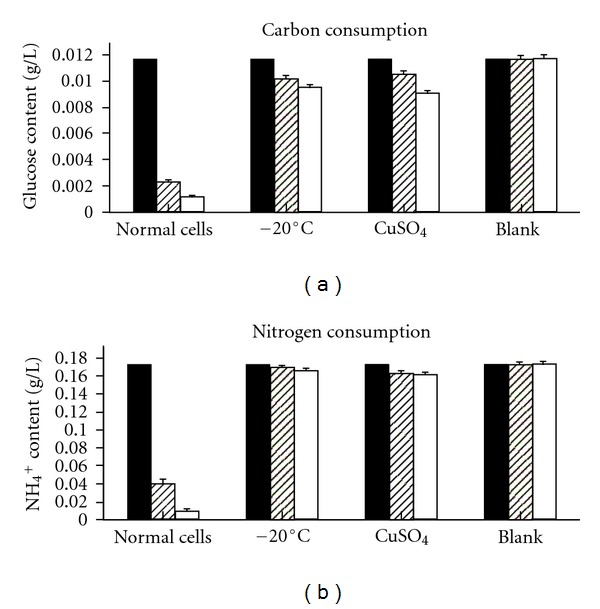
Metabolic activities of normal cells and VBNC* S. typhi* cells (induced by −20°C temperature and CuSO_4_). Glucose content at the baseline time point (black bars above). Glucose content after 24 h (grid bars above). Glucose content after 48 h (white bars above). NH_4_^+^ content at the baseline (black bars below). NH_4_^+^ content after 24 h (grid bars below). NH_4_^+^ content after 48 h (white bars below).

Both VBNC and normal cells were cultured in liquid medium at 150 rpm at 37°C for 48 h. As shown in Figure 3, the concentration of (NH_4_)_2_SO_4_ and glucose were measured by ion chromatography and Fehling's solution, respectively. In normal cells, approximately 74% glucose (carbon source) was consumed after 24 h and 77% after 48 h, compared to 80% of the (NH_4_)_2_SO_4_ (nitrogen source) lost after 24 h, and 90% after 48 h. In VNBC cells, glucose was reduced by only 3%–5%, and the (NH_4_)_2_SO_4_ was reduced by approximately 13%–19%. Normal cells consume more carbon and nitrogen sources than VBNC cells, but the decrease in the carbon and nitrogen sources for the VBNC cells indicated that they remained viable.

### 3.4. Resuscitation

Nutrient broth was used to resuscitate the VBNC cells of *S. typhi*. The VBNC cells induced by −20°C or CuSO_4_ were incubated with a temperature upshift for about 7 days, but the culturable populations were not found on the nutrient broth which suggested that resuscitation by temperature upshift still need a further study. However, VBNC cells were incubated in the nutrient broth, and the culturable populations were found when 1% (w/v) of catalase or 3% (v/v) of Tween 20 was added to the liquid broth medium for 24 or 48 h at 37°C, respectively. No microorganism was cultured from the broth medium of the control.

### 3.5. Virulence Assay

Thirty healthy Kunming mice were randomly divided into three groups for the virulence study. The control group was administered a lung lavage of autoclaved saline water, while the other two groups were administered a lung lavage of either VBNC cells or resuscitated cells for 3 days. All mice of the other two groups developed diarrheal illness, but none of the mice in the control group became unwell. Culturable *S. typhi* cells were isolated from the ascites fluid of these two groups.

## 4. Discussion

Nowadays, many people believe that bottled drinking water contains fewer contaminants, and they also dislike the taste of chlorinated tap water [35]. Bottled drinking water is becoming perceived as a healthier choice than tap water. However, this water source may be associated with health risks worldwide [36], as some VBNC-state pathogenic bacteria may exist in bottled drinking water that are exposed to copper-based biocide and are subjected to low temperatures during storage.* S. typhi* has been identified as the pathogen in drinking water outbreaks [37]. The large bottled drinking water consumption in the world may have increased the risk of disease.

Since the VBNC state was proposed, more than 60 species have been demonstrated to enter the VBNC state [25, 38]. The environmental conditions required to enter the VBNC state differ between bacterial species. Various bacteria can enter the VBNC state in low temperature environments, including *Vibrio parahaemolyticus* [31], *Aeromonas hydrophila* [39], and *Yersinia pestis* [40], amongst others. Subsequently, more reports regarding the induction of the VBNC state induced by CuSO_4_ have appeared [15, 41]. In the present study, as with many other Gram-negative bacteria, starved *S. typhi* cells could be induced into a VBNC state by either incubation at −20°C or by 0.01 or 0.015 mmol/L CuSO_4_.

The VBNC cells were shown to be decreased in size and were coccoid in shape compared to the normal rod-shaped cells, but the metabolic activity of the dormant cells still existed. Cells elongated as soon as a nutrition source was added. The ability to be resuscitated is an important characteristic of VBNC cells if they are placed in appropriate conditions. The resuscitation of *S. typhi* cells in this study was achieved after Tween 20 or catalase was added. Tween 20 is a good surfactant that may reduce surface tension and ameliorate the relation between VBNC cells and the culture medium [42]. Tween 20 can also be used as a carbon source that supplies energy to VBNC cells [43]. Catalase can reduce the levels of peroxide that may be generated when VBNC cells are resuscitated, which can cause cell injury [44].

Studies regarding VBNC bacteria may play an important role in human and animal health planning, as these dormant cells retain their infectious and pathogenic potentials [45]. A greater understanding of VBNC-state cells may help us to explain why the number of bacterial infections are reduced in winter [46]. It may also enable the discovery of new species in the sea or soil. Finally, the VBNC state could be useful as a method for bacterial culture collection.
